# Effect of Bran Pre-Treatment with Endoxylanase on the Characteristics of Intermediate Wheatgrass (*Thinopyrum intermedium)* Bread

**DOI:** 10.3390/foods10071464

**Published:** 2021-06-24

**Authors:** Yaxi Dai, Radhika Bharathi, Jacob Jungers, George Amponsah Annor, Catrin Tyl

**Affiliations:** 1 Department of Food Science and Technology, University of Georgia, Athens, GA 30602, USA; yaxidai@uga.edu; 2 Department of Food Science and Nutrition, University of Minnesota, Saint Paul, MN 55108, USA; bhara056@umn.edu (R.B.); gannor@umn.edu (G.A.A.); 3 Department of Agronomy and Plant Genetics, University of Minnesota, Saint Paul, MN 55108, USA; junge037@umn.edu

**Keywords:** intermediate wheatgrass, arabinoxylans, TAXI, xylanase, bran treatment, bran-enriched bread, perennial grains

## Abstract

Previous work indicated that bran removal promotes network formation in breads prepared from intermediate wheatgrass (IWG) flour. However, refinement reduces yields as well as contents of nutritionally beneficial compounds such as fiber. This study evaluated xylanase pretreatment of IWG bran as a processing option to enhance the properties of bread made with half of the original bran content. Xylanase pretreatment did not affect stickiness but significantly reduced hardness and increased specific loaf volumes compared to negative (without xylanase) and positive controls (with xylanase but without pretreatment). However, the surface of breads with pretreated bran was uneven due to structural collapse during baking. Fewer but larger gas cells were present due to pretreatment. Addition of ascorbic acid modulated these effects, but did not prevent uneven surfaces. Accessible thiol concentrations were slightly but significantly increased by xylanase pretreatment, possibly due to a less compact crumb structure. Endogenous xylanases (apparent activity 0.46 and 5.81 XU/g in flour and bran, respectively) may have been activated during the pretreatment. Moreover, Triticum aestivum xylanase inhibitor activity was also detected (193 and 410 InU/g in flour and bran). Overall, xylanase pretreatment facilitates incorporation of IWG bran into breads, but more research is needed to improve bread appearance.

## 1. Introduction

Whole grains and products thereof are integral parts of diets that are not only healthy but also have low environmental impact [1]. For instance, in a recent study, participants who adhered to a diet with a low index for healthiness and sustainability consumed significantly fewer whole grains than individuals whose diet scored high on both aspects [2]. Indigestible carbohydrates that qualify as dietary fiber are considered one of the main contributors to the positive health effects associated with the consumption of whole grains and cereal brans. For example, cereal fiber intake has been linked to a risk reduction for certain types of cancers, mainly of the digestive tract [3], or cardiovascular diseases [4]. However, the incorporation of cereal bran into food products can decrease consumer acceptance and the decline in acceptability with increasing fiber levels appears to be especially pronounced for indulgence products [5]. In baked goods, the lower acceptance may be at least partly attributable to structural and textural changes induced by the presence of cereal fiber [5]. Thus, numerous studies have focused on devising novel processing strategies for cereal bran such as particle size reduction or fermentation [6]. Such approaches may be particularly valuable for raw materials with high insoluble dietary fiber contents, such as the perennial grain intermediate wheatgrass (IWG). This relative of wheat and rye has recently become commercially available on a small scale and is the subject of domestication efforts aimed to develop an alternative to annual grains [7]. Its cultivation markedly differs to that of annual grains in its effect on parameters related to water and soil health, e.g., leading to about two orders of magnitude less nitrate leaching than corn cultivation [8]. However, using IWG flour in baking applications is associated with several challenges that relate to its composition, specifically a lack of high-molecular weight glutenins [9,10] and higher insoluble fiber contents (17–21% in whole grain IWG [11]) than present in bread wheat. To overcome these challenges, previous studies evaluated the effect of commercially available endoxylanase on IWG dough [11] and bread [12] properties, made from completely, partially (containing 50% of the original bran), or unrefined flour. Use of such an enzyme has been shown to enhance the properties of wheat breads if water-unextractable arabinoxylans (WU-AX) are cleaved [13]. Similar effects were observed in breads made from other flours, e.g., xylanase addition to rye [14] and spelt bread [15] increased the loaf volume. However, the only positive effect observed in IWG breads was a minor increase in specific loaf volume, which only occurred in breads from refined flour. The addition of ascorbic acid had a marked effect on crumb structure and surface appearance [12], but had no effect on loaf volume. The positive effects of ascorbic acid were only observed in bread from refined flour and in one sample containing 50% of the bran.

Arabinoxylans (AX) isolated from insoluble IWG fiber have been shown to have an arabinose to xylose ratio between the values reported for rye and wheat [16]. Side chains were short and sparse, which may facilitate glycosidic cleavage by endoxylanases, as these enzymes avoid branching points [13]. The effect of xylanases on dough and bread properties is influenced by numerous factors such as the dosage used but also the presence of xylanase inhibitors in the flour [17]. In addition, product properties may only be improved if WU-AX present in bran layers are specifically targeted. In this study, we assessed the effect on volume, texture and crumb structure exerted by separate pretreatment of bran with xylanase before adding back 50% of the original bran content to endosperm flour. This study’s design included a negative control without xylanase as well as a positive control, i.e., xylanase directly added to the blend of endosperm flour and bran without pretreatment. In addition, we evaluated whether the addition of ascorbic acid to breads made with xylanase (either with or without pretreatment) would have any synergistic effect on the tested bread properties. The overall aim of the study was to determine whether processing operations can modify bran properties sufficiently to allow for inclusion into bread recipes without diminishing product quality. Such products could appeal to consumers who are interested in following a diet intended to foster their own health as well as that of the planet.

## 2. Materials and Methods

### 2.1. Materials

Grains of the IWG variety ‘MN-Clearwater’ were collected from three production fields located in Rosemount, Cold Spring, and Cannon Falls (MN, USA), and planted in September of 2018. Grain was obtained in 2020 by removing random samples from the combine hopper after harvest, dried on the same day, and dehulled before milling. Chemicals were obtained from Fisher Scientific (Waltham, MA, USA) and MilliporeSigma (St. Louis, MO, USA) and of reagent grade or higher. Ascorbic acid was purchased from Duda Energy (Decatur, AL, USA). Xylanase (PowerBake 960, 90,623 units/g, exhibiting 510 units/g α-amylase side activity) was donated by DuPont (Wilmington, DE, USA). The activity of endogenous endo-1,4-β-D-xylanase in flour and bran was determined via the Xylazyme AX assay kit acquired from Megazyme (Bray, Ireland). 

### 2.2. Preparation of Flour and Bran

Dehulled IWG kernels (1.122 kg) were tempered and milled as described previously [18] on a Brabender Quadrumat Junior mill (C.W. Brabender Instruments, Hackensack, NJ, USA), refined flour obtained by sieving (particle size < 250 μm), and bran after milling to the same particle size using a Udy Cyclone Sample Mill (Udy Corporation, Fort Collins, CO, USA). The ratio of endosperm flour to bran was 55.3:44.7. For breadmaking, a flour to bran ratio of 77.7:22.3 (Table 1) was used, corresponding to 50% of the original bran. This amount was chosen because previous work [11,12] had indicated that above a certain threshold bran restricted the effectiveness of conditioners. Some studies on wheat bread have also used bran addition in this range, e.g., 8–40% based on flour weight [19,20]. Additionally, some authors have concluded that bran contents should not exceed 50% of the flour weight in bread-making (assuming minimal textural and structural changes compared to breads from refined flour as the goal) [21].

### 2.3. Xylanase Treatment of Bran and Preparation of Breads

Treatment of bran was based on a previous method developed for wheat bran, with slight modifications [22]. Bran was combined with water (ratio of 1:3) in the absence or presence of 37.5 ppm xylanase, with the dosage based on previous studies [11,12]. After 90 min of incubation at 55 °C, the mixture was filtered over glass wool, the filtrate was discarded, and the pretreated bran weighed. Pretreated bran was then combined with refined IWG flour using a spatula. To this blend, an amount of water corresponding to the difference between the water used for pretreatment (51.66 g) and the total water needed (55.08 g, based on reported values for Farinograph water absorption [23]) was added. This was done to ensure that all doughs were prepared at an equal moisture level (61.2% on a flour basis). For control experiments without bran pretreatment (Table 1), bran and endosperm flour were mixed directly. In breads that contained ascorbic acid it was pre-blended with endosperm flour (85 ppm). 

Breads were prepared based on AACCI method 10–10.03 with slight modifications as reported previously [12]. Ingredients other than sugar and yeast in Table 2 were mixed by hand and a spatula to make dough.

### 2.4. Determination of Arabinoxylans, Apparent Endogenous Xylanase Activity and Xylanase Inhibition 

For analysis of total AX, samples were hydrolyzed (20 ± 2 mg) with 1 M HCl (5 mL) at 90 °C for 1 h. After neutralization with 1 M NaOH, the samples were diluted (1:4 for flour, 1:10 for bran), filtered through 0.45 µm syringe filters, and analyzed via High-Performance Anion Exchange chromatography using a Dionex ICS 3000 HPAEC system (Dionex Corporation, Sunnyvale, CA, USA) with a pulsed amperometric detector. A CarboPac PA100 (Thermo Fisher) column (250 × 4 mm) and guard column (50 × 4 mm) and injection volumes of 250 µL were used. Eluents and operating conditions were based on literature [24]. Water-extractable arabinoxylans (WE-AX) were analyzed on the same system with the same set-up, but obtained from flour (200 mg) or bran (100 mg) by incubating samples with 10 mL water at 30 °C for 2 h, centrifuging at 2500× *g* for 10 min then transferring aliquots (2 mL) of the supernatant to a fresh tube and then hydrolyzing them using the same steps described above for total AX. AX contents were calculated as the sum of arabinose and xylanose, determined via external standard curves, and multiplied by 0.88 [24].

Endogenous xylanase activity and xylanase inhibition by Triticum aestivum xylanase inhibitor (TAXI) were measured based on a previous method [17], with modifications to reduce sample size. Samples (100 mg flour or 50 mg bran; *n* = 4) were combined with 1 mL of 25 mM pH 4.7 sodium acetate buffer for the analysis of xylanase activity. For TAXI analysis, a *Bacillus subtilis* xylanase solution, flour or bran were combined with sodium acetate buffer (25 mM, pH 4.7 containing 0.5 g/L bovine serum albumin), then samples were shaken at room temperature for 1 h. After centrifugation (9600× *g*, 10 min), 0.5 mL of the supernatant were transferred to a test tube and incubated at 50℃ for 5 min. This was followed by the addition of a Xylazyme-AX substrate tablet and 17 h of incubation at 50 °C before addition of 1% Trizma base (10 mL). Then, the mixture was filtered through Whatman #1 filter paper before measuring A_590_ of the filtrates on a spectrophotometer against a blank prepared from sodium acetate buffer. The flour and bran extracts were appropriately diluted to ensure the results fell in the range of a standard curve prepared with *Aspergillus niger* xylanase (part of the Megazyme kit). Results were expressed according to Gebruers et al. [17] with 1 xylanase unit (XU) representing the amount of enzyme required to raise the A_590_ by 1.0 per hour of treatment and 1 inhibitor units (InU) denoting the amount of inhibitor that can inhibit 50% of xylanase activity. Moisture in flour and bran was assessed (*n* = 3) via AACC 44–15.02 [25] to express values on a dry matter (d.m.) basis. 

### 2.5. Stickiness

A Chen-Hoseney stickiness rig (Texture Technologies, Hamilton, MA, USA) and a TA.XT-Plus Texture Analyzer (Texture Technologies) were used to measure stickiness (N) of dough samples in triplicate. About 3 g of dough were put into the chamber of the dough stickiness cell and extruded through the die. The first 1 mm of dough on the screw cap was discarded. Another 1 mm of dough was extruded, rested for 30 s and then stickiness was measured using a trigger force of 5 g, a 5 kg load cell, and an applied force of 40 g.

### 2.6. Bread Analysis

Loaf volume (*n* = 3), hardness and resilience (*n* = 6) were assessed according to AACC methods 10–05.01 and 74–09.01. For hardness and resilience a TA.XT-Plus Texture analyzer with TA-5 attachment, a cylindrical probe of 1.3 cm diameter and 3.5 cm length were used. Cell counts, average cell size and total cell area in crumb (*n* = 4) were analyzed with ImageJ version 1.50i (National Institute of Health, Rockville, MD, USA) after converting scanned images with Otsu’s auto-threshold, based on a previous study [12].

### 2.7. Accessible and Total Thiols

The accessible thiol concentration in dough (frozen with liquid nitrogen and then freeze-dried) was measured according to a previous study [26]. A 20 mg portion of sample was shaken with 1 mL of 0.05 M sodium phosphate (pH 7.0) containing 0.1 M NaCl and 0.5 mM 5,5′-dithiobis (2-nitrobenzoic acid for 1 h at room temperature. After centrifugation (11,000× *g*, 3 min) the A_412_ of the supernatant was determined. An extinction coefficient ε of 14,150 M^−1^ cm^−1^ [27] was used to express results. For determination of total thiols, the same method was used but the reagents supplemented with 1% sodium dodecyl sulfate. Protein contents of dough samples were quantified according to AACC 46–30.01 on a FP828 Leco nitrogen analyzer (Leco corporation, St. Joseph, MI, USA) using a nitrogen factor of 5.7 and thiol data reported in µmol/g protein. 

### 2.8. Statistical Analysis

Three replicates of dough and bread samples were prepared and analyzed at least in triplicate. One way analysis of variance (ANOVA) was done using R (version R 3.6.1,R Core Team, Vienna, Austria) and differences among means were determined using the Tukey-Kramer Honest Significant Difference (HSD) test at *p* ≤ 0.05. Differences between flour and bran in arabinoxylan contents and their respective activities of endogenous xylanase and TAXI were analyzed via a *t*-test, after assessing homogeneity of variances with an F-test. Model assumptions were verified by examining diagnostic plots of model residuals.

## 3. Results and Discussion

### 3.1. Specific Loaf Volume

**Figure 1:**
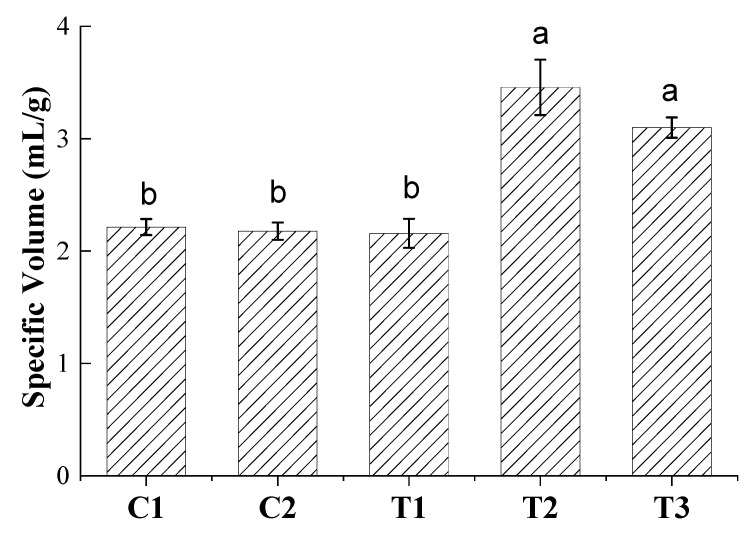
Specific volume of IWG breads (*n* = 3) as affected by bran treatment with xylanase and use of ascorbic acid. Different letters signify statistical differences (*p* < 0.05) among treatments.

The specific loaf volumes of IWG breads without pretreatment (C1, C2, T1) were 2.16–2.2 mL/g (Figure 1) and did not significantly differ from each other. These values were similar to previous results [12] on bread containing 50% of the original bran and either no dough conditioner or ascorbic acid (2.07–2.23 mL/g). IWG breads are dense loaves, as is often observed for rye breads [14] for which similar specific loaf volumes have been reported [28]. IWG breads without pretreatments had specific loaf volumes slightly below the range reported for breads made from whole wheat (2.43–3.64 mL/g) or spelt (2.94–3.87 mL/g) [15]. A slight but significant increase in specific loaf volume occurred in our previous work when xylanase was incorporated into dough made from refined IWG flour (2.54–2.85 mL/g), but this was not observed for breads containing 50% of the bran [12]. Some authors found that addition of xylanase increased specific volumes of breads made from refined or whole grain wheat [29] while in other studies it did not have an effect on the loaf volume of whole wheat breads [30,31]. These discrepancies may be due to differences in flour types and constituents as well as the xylanase types and dosages used.

Compared to breads without pretreated bran (C1, C2, and T1), the specific loaf volume of IWG breads was significantly (*p* < 0.05) increased if bran was pretreated with xylanase (T2 and T3) and elevated to values reported for breads from domesticated grains such as spelt [15] or blends of refined wheat flour with wholegrain flour or bran [19]. Thus, IWG bread volumes were affected similarly to wheat by bran pretreatment [32]. AX hydrolysis catalyzed by xylanase may decrease the competition for water between AX and proteins involved in network formation, as shown for wheat [33]. Previously it was observed that IWG doughs contain more β-sheets and fewer β-turns when bran is part of the system [11], which suggests that IWG bran has a dehydrating effect on IWG protein networks as described for wheat dough [34]. In rye, WE-AX are assumed to contribute to the stability of dough and may form networks that support protein networks [35]. Currently, little is known about the role of individual flour polymers in establishing networks in IWG dough, but conversion of bran AX by the xylanase used in this study appears to be conducive to dough expansion (Figure 1). 

### 3.2. Crumb Hardness and Resilience

**Figure 2:**
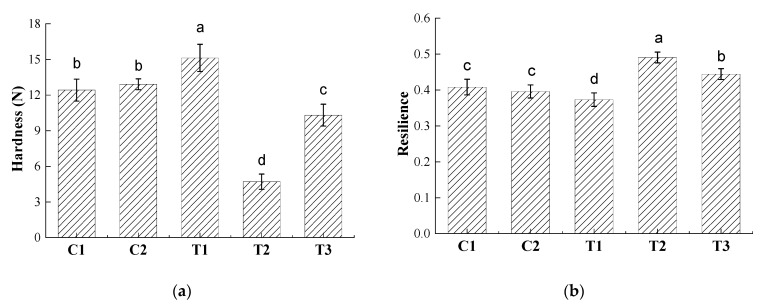
Hardness (**a**) and resilience (**b**) of breads with different treatments with different letters denoting statistical differences (*p* < 0.05) among treatments (*n* = 6).

Bran addition can reduce gas retention and thus decrease loaf volume and increase crumb hardness [20,29]. IWG breads C1, C2, and T1 exhibited high hardness (12.4 to 15.1 N), similar to bread from rye (9.6 to 14.4 N) [14] and above literature values for whole wheat bread [36]. However, as a consequence of the higher bran to endosperm ratio in IWG than in wheat kernels, partially refined IWG flours contain equal or higher insoluble dietary fiber than whole wheat [11]. Hardness was significantly (*p* < 0.05) lower in both (T2, T3) breads containing pretreated bran (Figure 2a), in line with findings for wheat bread [22]. Resilience of breads with pretreated bran was also significantly higher than when bran was not pretreated (Figure 2b). A study on bread made with different spelt varieties reported that ascorbic acid addition exerted a significant negative effect on resilience for one of the analyzed cultivars [15]. The same result was also observed for our samples (Figure 2b), for breads with (T3 vs. T2) or without bran pretreatment (T1 vs. C2). Wheat doughs are strengthened and stiffened by ascorbic acid [37], and we presume that in our study, it partially counteracted dough softening mediated by xylanase pretreatment. Future studies could evaluate if bran pretreatment and ascorbic acid addition affect texture over storage, as shown in a study on wheat bread where bran pretreatment reduced hardness of not only fresh but also stored breads [22].

### 3.3. Crumb Cell Structure

**Figure 3:**
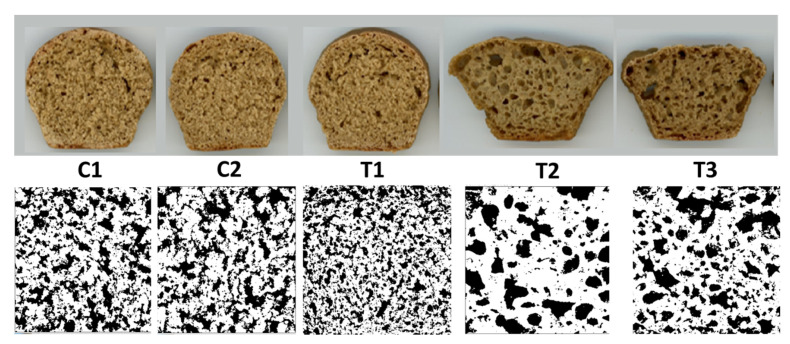
Slices (**top row**) and crumb scans (**bottom row**) of IWG bread formulated without pretreated bran (C1), without pretreated bran but with added xylanase (C2) or ascorbic acid (T1), with xylanase pretreated bran as-is (T2) or with added ascorbic acid (T3).

No significant differences among crumb characteristics were detected in bread slices without pretreated bran (C1, C2, T1), as shown in Table 3. The lack of difference in the area covered by cells among these samples is in agreement with previous results for bread made from partially refined flour [12]. In contrast, samples with pretreated bran (T2, T3) had significantly (*p* < 0.05) lower cell counts, higher average cell size and more area covered by cells than samples without pretreatment. Thus, the xylanase pretreatment improved gas holding, resulting in crumb that was less dense and contained fewer but larger cells (Figure 3). That a significantly higher percent of the area was covered by cells (Table 3) reflects the increased specific loaf volumes exhibited by T2 and T3 (Figure 1). In previous work on wheat [38], a significant negative correlation between specific loaf volume and hardness was reported. Crumb cells became less uniform upon xylanase addition in a dose-dependent manner. This was taken as an indication of a less dense crumb due to more voluminous and porous loaves, hence the decrease in uniformity.

The crumb of breads made with pretreated bran and ascorbic acid (T3) had significantly higher cell counts and average cell size than all breads without pretreated bran (C1, C2, T1), but lower values than crumbs in bread from pretreated bran without ascorbic acid (T2). Ascorbic acid therefore improved crumb porosity compared to control samples but limited the effect of bran pretreatment on the crumb to some degree.

Even though addition of ascorbic acid to bread without bran pretreatment (T1) did not increase the specific loaf volume, it exerted a different desirable effect by smoothening the crust (Figure 3). Such a result is in line with studies on wheat bread [39] in which ascorbic acid promotes disulfide bond formation and thereby strengthens networks [40]. We previously [12] observed the same effect in bread made from refined IWG flour from both growing locations but only in partially refined flour from one growing location, which corresponded to the sample with less total dietary fiber. Dough conditioners with a strengthening effect on protein networks are frequently combined with xylanase [39]. However, crust uniformity was only marginally enhanced in T3, i.e., when ascorbic acid was part of the recipe for bread with xylanase-pretreated bran. Ascorbic acid was used at a relatively low concentration (85 ppm, the same as used in previous work [12]) and possibly this level was insufficient to overcome collapse during baking. Higher ascorbic acid concentrations in the upper range of dosages typically used in industry (up to 150–200 ppm) [41,42] and their effects on networks could be evaluated in future studies. Additionally, proofing times may need to be optimized for IWG breads with pretreated bran.

Wheat flour’s balanced gliadin and glutenin profile leads to strain hardening during late proofing and at early baking stages and this makes dough in thinner regions harder to deform than dough in thicker regions [35], which contributes to uniformly distributed gas cells. The fact that IWG bread appearance suffers from uneven surfaces may be related to a low strain hardening index, which however would need to be assessed in future studies that could also investigate how the parameter is impacted by dough conditioners. In addition, components present in the aqueous dough phase (“dough liquor”) may also play a role in the stability of the system (or lack thereof), as has been shown in work that contrasted interface compositions and characteristics in dough from various cereals [28].

### 3.4. Dough Stickiness

Xylanases may increase stickiness because the reaction products (WE-AX) have lower water-holding capacity than the intact substrate (WU-AX) [13] and this may limit the amount of xylanase that can be added to bread doughs [43]. However, none of our samples differed in dough stickiness (Table 4). Other studies involving refined wheat [44] as well as in refined IWG [12] detected a stickiness increase upon ascorbic acid addition. The discrepancy between results for (refined) wheat [44] and the IWG doughs in this study may also be due to differences in the amount of ascorbic acid used (2110 vs. 85 ppm). Optimal amounts of ascorbic acid are influenced by varietal differences among flours and also depend on product type [41]. However, in our previous study [12] stickiness was not increased when either ascorbic acid or xylanase were added to breads made from partially refined flour, as the inclusion of bran affected dough and bread properties more than dough conditioner use. The presence of bran has been observed to decrease dough stickiness in other work as well [20]. In comparison, dough stickiness reportedly increased after xylanase addition in systems solely based on wheat flour [45] as well as for wheat-rye composites [46]. Factors such as the types and dosages of used xylanases may have contributed to these differences, in addition to differences in molecular characteristics of AX. 

### 3.5. Characteristics Related to Arabinoxylans in IWG Flour and Bran

The interaction of endoxylanase with the AX population in IWG flour and bran can be assumed to be influenced by structural characteristics of the AX. While a detailed characterization and profiling were outside of the scope of this study (also due to limited sample amounts), IWG flour and bran were analyzed for their contents in total and WE-AX as well as the arabinose to xylose ratios thereof, as shown in Table 5. Total AX content in IWG flour was similar to rye flour [47] and higher than in wheat [48] while the arabinose to xylose ratio was similar to bread wheat and higher than in rye [47,48]. IWG flour contains a similar amount of WE-AX as winter wheat, for which a wider range than for other cereal flours was reported [48]. Based on the arabinose to xylose ratio, WE-AX in IWG flour are less branched than those in wheat or rye [47,48]. As for IWG bran, its total arabinoxylan content was in the upper range of values reported for bread wheat [48] and their arabinose to xylose ratio was similar to the ratio of 0.36 reported for AX in insoluble fiber present in IWG whole meal [16]. The bran contained more WE-AX than found in wheat or rye, and they were similarly branched than bread wheat [47,48]. It is well-documented in literature that endoxylanases favor substrates with fewer branches [13]. The results in Table 5 suggest that the majority of IWG AX are only scantly substituted, which may promote their hydrolysis by endoxylanases, while the densely substituted WE-AX in IWG flour and bran may represent unfavorable substrates. This does not explain the apparent lack of effect on bread characteristics for the positive control. Further work is needed to elucidate if, e.g., the high water content during pre-treatment promoted mobility and thus access to the substrate for the enzyme, due to the plasticizing effect of water [49]. In addition, the fine structures of AX as impacted by xylanase treatment need to be assessed. 

Aside from the xylanase added as dough conditioner, endogenous endoxylanase may have been activated during bran pretreatment as observed after prolonged wheat bran hydration [50]. In line with reports for other cereals [17,51], apparent xylanase activity in IWG was significantly higher (*p* < 0.05) in bran than in endosperm flour (Table 5). The higher activity in cereal brans than in flours has been linked to the presence of microorganisms on kernel surfaces [17]. IWG breads supplemented with xylanase were also observed to have darker color than breads without xylanase addition. The results of IWG bran are above the values detected in some other cereal brans, such as spelt and rye, while the values for flour are in the range of barley and oat [17]. IWG is a hulled grain, like barley and oat, and the presence of hulls may promote microbial growth [17]. While the grains were dehulled prior to milling, there was a period of several weeks during which the hulls remained on grains; however, the grains were dried on the same day they were harvested. Moreover, the apparent xylanase activity results may be related to harvest methods used for the IWG samples. Grain was harvested by direct combining in two of the locations and was harvested from swathed windrows in the third. In studies on other cereal grains, high apparent xylanase activities in durum wheat were related to preharvest sprouting [17], and this may have also occurred in swathed plant material. Swathing has numerous advantages, such as a more flexible harvest time as well as reduction of kernel moisture if harvested at the correct time. However, it may increase vulnerability to microbial growth if windrows are rewetted via precipitation or heavy dew.

Most cereal grains exhibit high inhibiting activity against microbial xylanases, which is assumed to be part of plant defense mechanisms [52], but has the consequence of potentially counteracting the effect of xylanases added as processing aids [17,53]. Three proteins with xylanase inhibiting ability have been described in cereals, and they target xylanases of different origin. Xylanases belonging to family 11 of glycoside hydrolases, such as the xylanase used in this study, are inhibited by TAXI [54]. The activities of TAXI in IWG endosperm flour and bran (Table 5) were at the upper end of the range reported for other cereal grains, such as bread wheat and rye [17]. To the best of our knowledge, the values reported in Table 5 are the first published assessments for apparent xylanase and TAXI activity in IWG flour and bran. Work on other cereals has highlighted the influence of genetic and environmental factors (and their interactions) on these parameters [53] and thus further studies are needed, using a larger set of IWG samples cultivated under different conditions, to better understand their impact on end use properties. Studies would also need to assess at what point during bread preparation the inhibitors influence added microbial xylanase the most as well as whether different kinds of AX are preferably hydrolyzed at different processing stages. Work that used refined wheat flour [43] has indicated that despite of being susceptible to inhibition by some inhibitors, some xylanases were active until the first punching step during breadmaking and it was hypothesized that xylanase inhibitors were initially associated with AX and thereby inactivated. This was also observed in a previous study [55] which showed that inhibition of the employed endoxylanase reduced stickiness and impacted characteristics related to WE-AX more than those linked to WU-AX solubilization. 

### 3.6. Effect of Pretreatment and Ascorbic Acid on Thiol Groups in Dough

**Figure 4:**
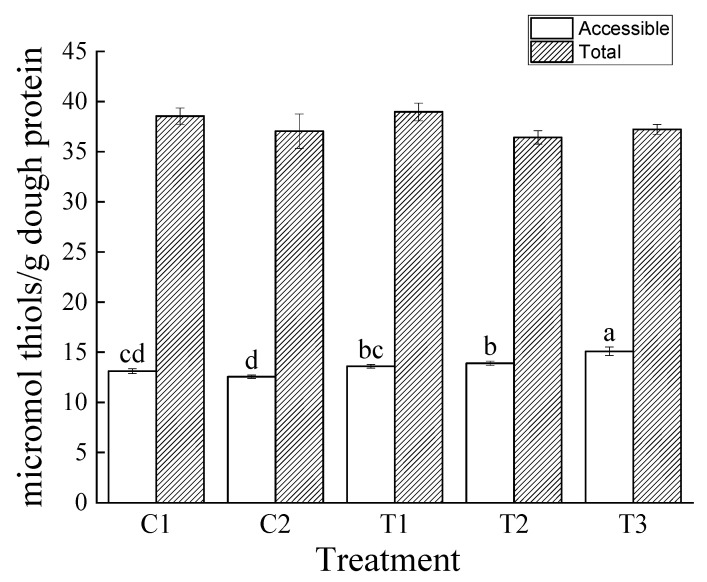
Accessible (white columns) and total (striped columns) thiols in dough samples. For sample identifiers, see Table 1. Error bars represent standard deviations (*n* = 3) and different letters denote significant differences among accessible thiol samples. No significant differences were present among total thiols.

In wheat, thiol groups are crucially involved in the formation of protein networks as well as their modification via disulfide exchange reactions, and their evolution over processing may reflect changes in network compactness [27]. As shown in Figure 4, the concentrations of accessible thiols were significantly (*p* < 0.05) higher in doughs prepared with pre-treated bran as compared to the positive and negative control. In contrast, total thiols were not different among samples, and similar to the value reported for wholegrain IWG flour [56]. On the other hand, accessible thiol concentrations were higher than previously observed in IWG flour [56], which is in line with findings for wheat dough for which it was assumed that hydration leads to exposure of thiols previously buried inside proteins [34]. Albumins and globulins, which represent main protein fractions in cereal bran layers [57], are rich in cysteine and thus thiol groups and may get incorporated into protein networks [58]. In addition, thiol groups occur in the form of glutathione, a tripeptide known to depolymerize gluten networks in wheat [59]. A negative relationship has previously been observed for higher flour concentrations of accessible thiols and specific loaf volume of breads thereof [34] and fewer thiols may indicate a more extended gluten network. This was not the case in our sample set, and xylanase pre-treatment increased thiol accessibility as well as loaf volume. A similar result was obtained for whole wheat gluten to which xylanase was added [60]. Moreover, the accessible thiol concentrations were affected by flour particle size [60] and future work could assess how particle size (distribution) of IWG influences network formation in general and the effect of enzymatic treatments in particular. The fact that the highest concentration of accessible thiols was found for dough made with pre-treated bran and ascorbic acid was unexpected, as its oxidation product dehydroascorbic acid can oxidize and thereby remove glutathione [40]. However, molecular oxygen is needed in order to exert this effect [40], and the mixing conditions in our study (samples were mixed by hand) may not have been well-suited to promote the reaction. Moreover, the dough samples were only analyzed at one time point, and thus follow-up studies could evaluate the progression of accessible thiols and other parameters related to network formation over the processing steps. Little information is known about how ascorbic acid affects protein networks in non-wheat systems, and it may be interesting to compare and contrast the effect of bran pretreatment and ascorbic acid addition on dough made from wheat vs. IWG.

## 4. Conclusions

The outcomes of this study indicate that successful incorporation of IWG into breads is not restricted to the use of refined flour if bran is suitably processed. Bran pretreatment with endoxylanase affected the appearance and texture of bread by increasing the specific loaf volume, resilience and gas cell size while decreasing hardness and the number of gas cells. The same endoxylanase, used at the same concentration, did not exert the same effects when added to bran that was mixed with refined flour. The low degree of branching in bran WU-AX may have facilitated their hydrolysis by the enzyme. However, further work is needed to understand the collapse occurring after proofing as well as finding strategies to prevent it.

Similar to a previous study, surfaces of IWG breads were rugged in controls, but addition of ascorbic acid to breads made without pretreatment made the surfaces smoother. To some degree, ascorbic acid modulated the effects of xylanase in pre-treated breads. Upon ascorbic acid addition, loaves with pre-treated bran became harder and denser, suggesting enhanced cross-linking. However, an increase in thiol groups was observed, which necessitates detailed studies on protein networks to evaluate if ascorbic acid affects cysteine residues in a similar way as it does in wheat. In addition, future studies could analyze dough and bread samples at different time points over processing and also determine glutathione levels to provide insights on the interaction of ascorbic acid with IWG dough constituents.

The high endogenous xylanase activity may have been linked to harvest techniques. Agronomic studies are crucial for providing farmers with guidance on how to best grow this novel crop. However, our results highlight that management practices during IWG cultivation and harvest may affect end-use properties, which underlines the importance of continuing collaborations between plant and food scientists for further development of this novel crop.

## Figures and Tables

**Table 1:** Formulation of intermediate wheatgrass bread. Plus signs (+) denote the presence, minus signs denote (−) the absence of a conditioner or treatment.

Treatment	Ratio of Flour to Bran	Pretreatment	Xylanase	Ascorbic Acid
C1	77.7:22.3	−	−	−
C2		−	+	−
T1		−	−	+
T2		+	+	−
T3		+	+	+

**Table 2:** Ingredients incorporated into intermediate wheatgrass dough.

Ingredient	Amount (g)
Flour	90
Sugar	5.40
Salt	1.35
Yeast	4.77
Shortening	2.70
Water	55.08

**Table 3:** Effect of bran incubation and ascorbic acid addition on stickiness of intermediate wheatgrass dough.

Treatment ^1^	Cell Counts (Cells/cm^2^)	Average Cell Size (cm^2^)	Area Covered by Cells (%)
C1	60 ± 11 ^a^	0.011 ± 0.0052 ^c^	62.8 ± 1.8 ^b^
C2	58 ± 11 ^a^	0.012 ± 0.0010 ^c^	62.7 ± 1.5 ^b^
T1	59 ± 4 ^a^	0.011 ± 0.0025 ^c^	63.4 ± 1.3 ^b^
T2	25 ± 6 ^c^	0.028 ± 0.0028 ^a^	69.2 ± 2.9 ^a^
T3	40 ± 4 ^b^	0.017 ± 0.0028 ^b^	66.5 ± 1.6 ^a^

^1^ Values within a column that are followed by a different letter differed significantly from each other. Sample identifiers are listed in Table 1.

**Table 4:** Effect of bran incubation and ascorbic acid addition on stickiness of intermediate wheatgrass dough (*n* = 3).

Treatment ^1^	Stickiness (N)
C1	0.164 ± 0.029
C2	0.152 ± 0.075
T1	0.189 ± 0.020
T2	0.139 ± 0.056
T3	0.195 ± 0.064

^1^ For sample identifiers see Table 1.

**Table 5:** Contents and arabinose to xylose ratio (A/X) of arabinoxylan (AX) populations, and activities of endogenous xylanase and *Triticum aestivum* xylanase inhibitor (TAXI) in refined flour and bran from intermediate wheatgrass on a dry matter (d.m.) basis.

Sample	Total AX (% of d.m.) ^1^	A/X in Total AX ^1^	Water-Extractable AX (% of d.m.) ^1^	A/X in Water-Extractable AX ^1^	Apparent Xylanase Activity (XU/g of d.m.) ^1^	TAXI Activity (IU/g of d.m.) ^1^
Refined flour	3.73 ± 0.15 ^b^	0.569 ± 0.002 ^a^	0.94 ± 0.03 ^b^	1.004 ± 0.001 ^a^	0.46 ± 0.04 ^b^	193 ± 6 ^b^
Bran	19.94 ± 0.67 ^a^	0.401 ± 0.038 ^b^	1.68 ± 0.17 ^a^	0.873 ± 0.015 ^b^	5.81 ± 0.36 ^a^	410 ± 2 ^a^

^1^ Means in the same column followed by a different letter differ significantly (*p* < 0.05). Results are stated as averages ± standard deviation (*n* = 4) except for total and water-extractable arabinoxylans in flour where variability denotes half the range (*n* = 2).

## Data Availability

Data is contained within the article.
